# A 3D *Fusarium* keratitis model reveals isolate-specific adhesion and invasion properties in the *Fusarium solani* species complex

**DOI:** 10.1128/msphere.00328-25

**Published:** 2025-11-04

**Authors:** Anna Zimmermann, Johanna Theuersbacher, Hong Han, Léonie Herzog, Benedikt Schrenker, Christian Lotz, Christian Stigloher, Jost Hillenkamp, Kerstin Hünniger-Ast, Grit Walther, Daniel Kampik, Oliver Kurzai, Ronny Martin

**Affiliations:** 1 Institute for Hygiene and Microbiology, University of Würzburg 9190, Würzburg, Germany; 2 Department of Ophthalmology, University Clinic Würzburg 155918, Würzburg, Germany; 3 Translational Center Regenerative Therapies (TLC-RT), Fraunhofer-Institute for Silicate Research 28474, Würzburg, Germany; 4 Department of Functional Materials in Medicine and Dentistry, Institute of Functional Materials and Biofabrication, University of Würzburg 9190, Würzburg, Germany; 5 Imaging Core Facility of the Biocenter, Theodor-Boveri-Institute, Universitätsklinikum Würzburg 27207, Würzburg, Germany; 6 Research Group Fungal Septomics, Leibniz Institute for Natural Product Research and Infection Biology–Hans-Knoell Institute 28406, Jena, Germany; 7 National Reference Center for Invasive Fungal Infections, Leibniz Institute for Natural Product Research and Infection Biology–Hans-Knoell Institute 28406, Jena, Germany; Max-Planck-Institut fur Biologie Tubingen, Tubingen, Germany

**Keywords:** *Fusarium*, fungal keratitis, cornea infection model, fungal invasion

## Abstract

Members of the *Fusarium solani* species complex (FSSC) are major causes of keratitis in humans. The underlying mechanisms leading to this disease are not well understood, partially due to the absence of more complex *in vitro* infection models. Here, we compared the pathogenicity of keratitis-causing FSSC members *F. falciforme*, *F. keratoplasticum*, and *F. petroliphilum* in a 2D monolayer infection model using a human corneal epithelial cell line and a newly established 3D human cornea infection model which comprises the multilayer epithelium and the stroma. In both models, *F. keratoplasticum* emerged as the most virulent species, showing extensive invasion and host cell damage and, in rare cases, even inducing the formation of transcellular tunnels. While *F. falciforme* exhibited strong adhesion to corneal epithelial cells, its capacity for invasion and damage was limited. *F. petroliphilum* was the least virulent among the FSSC species with low adhesion and invasion. The use of the 3D model allowed the investigation of fungal infection in a more physiologically relevant context and revealed that all three FSSC species disseminated deeper into the cornea than *C. albicans* under identical conditions. This may partly explain the unfavorable clinical outcome of *Fusarium* keratitis in patients, as the deep penetration of *Fusarium* hyphae complicates the accessibility of antifungal drugs to the pathogen. Our data indicate species-specific infection routes which might avoid recognition by the host cell defense system and could contribute to the overall high virulence of the species.

**IMPORTANCE:**

*Fusarium* keratitis is a rare fungal infection of the human eye. The outcome for affected patients is often poor, with loss of eyesight or even the entire eye being common. Investigation of this disease is challenging due to the absence of established *in vitro* complex infection models that go beyond a simple 2D monolayer of a single cell type. Here, we performed a comparative analysis of three *Fusarium* species in a classic 2D infection model and a newly established 3D human cornea model which comprised the epithelium and the stroma. Our experiments revealed that *F. keratoplasticum* shows a higher potential for invasion and host cell damage when compared to related species. The 3D human cornea model could be a helpful tool for future investigations of fungal pathogenicity and antifungal drug susceptibility during cornea infections.

## INTRODUCTION

Fungal keratitis is a rare, yet challenging infectious disease. The annual global incidence is around one million cases (1). In Germany, one-third of the cases are caused by *Candida* species, while the remaining two-thirds are predominantly due to filamentous fungi. *Fusarium* species are causative for nearly half of these infections, followed by *Aspergillus* and further filamentous species (2). The diagnosis of mold keratitis is achieved late in many cases (3). Despite aggressive local and systemic treatment, the outcome is frequently poor (4). The disease often affects patients who are otherwise healthy, leading to severe visual impairment or even permanent blindness due to fatal damage of the ocular tissue (5). In many cases, surgical interventions are inevitable (6). In Europe, enucleation of the eye is required in approximately 7% to 9% of cases (7, 8). Globally, *Fusarium* is the most frequent mold keratitis pathogen (9). The genus possesses a high resistance toward common antimycotics (5). The high proportion of *Fusarium* keratitis cases, combined with the challenges in treatment, highlights the importance of *Fusarium* as a keratitis pathogen and underscores the need for continued research in this area.

The genus *Fusarium* comprises more than 300 species grouped into several species complexes (10, 11). The *Fusarium solani* species complex (FSSC) includes the most frequently isolated pathogens in human keratitis (2, 9), and it is debated to consider it as a separate genus named *Neocosmospora* (12–14). All clinically relevant FSSC members are assigned to clade 3, including the most common keratitis-causing ones: *F. keratoplasticum* (syn. *Neocosmospora keratoplastica*), *F. petroliphilum* (syn. *Neocosmospora petroliphila*), and *F. falciforme* (syn. *Neocosmospora falciformis*) (15–17). FSSC species are commonly not limited to one specific host kingdom. Environmental and phytopathogenic inhabitants can also act as opportunistic animal and human pathogens (17). To date, however, most of the evidence for virulence mechanisms in *Fusarium* comes from studies on plant infections. Species- and strain-specific accessory chromosomes, non-coding RNAs, and extracellular vesicles are known to play roles in *Fusarium* virulence, highlighting the high complexity of *Fusarium* pathogenicity (18–20). In contrast, insights into mechanisms governing human infection are scarce. The research on fungal keratitis is currently mainly based on animal models. While some studies involve *Fusarium* isolates, a comparison of different species and strains within the same species complex remains new (21).

Here, we analyzed infection properties of the FSSC species *F. falciforme*, *F. petroliphilum*, and *F. keratoplasticum*. The aim of our study was to determine whether (i) we can detect significant differences in adhesion, invasion, and tissue damage between the three closely related species and (ii) a complex 3D infection model is advantageous for studying *Fusarium* keratitis infection.

## MATERIALS AND METHODS

### Fungal strains and growth conditions

The clinical isolates of *F. falciforme* (NRZ-2015-096, NRZ-2016-123, NRZ-2020-612, NRZ-2019-673), *F. petroliphilum* (NRZ-2014-013, NRZ-2014-079, NRZ-2017-410, NRZ-2018-201), and *F. keratoplasticum* (NRZ-2014-069, NRZ-2017-556, NRZ-2016-116, NRZ-2021-330, NRZ-2021-275) were obtained from the German National Reference Center for Invasive Fungal Infections (NRZMyk). All strains were routinely grown on Sabouraud agar plates for three to days at 28°C. *C. albicans* SC5314 (22) was used as control and grown for 24 h at 37°C on yeast extract peptone dextrose (YPD) agar plates. For infection assays, *Fusarium* microconidia were harvested in sterile water and quantified using a Neubauer cell counting chamber.

### Epithelial cell line culture

The telomerized (hTERT immortalized) cell line hTCEpi (Evercyte) was cultivated in KBM-2 Medium (Lonza) at 37°C with 5% CO_2_. They were either used to cultivate monolayers on 48-well plates or coverslips coated with collagen I (Advanced BioMatrix) in 24-well plates, with 4 × 10^4^ and 1 × 10^5^ cells per well, respectively. The cells were subsequently grown to confluence within one or three days. Alternatively, 5 × 10^5^ cells were seeded directly onto the compressed 3D model stromal layer, as described below.

### 3D model assembly

The hemi-cornea 3D model consists of a multilayered epithelium of hTCEpi cells and a stromal layer composed of collagen and primary human fibroblasts. The models were built in 6-well polycarbonate snap-transwell plates with membrane inserts having a pore size of 0.4 µm (Corning). As an initial step, 1.8 × 10^6^ primary human fibrocytes (isolated from donor corneas that failed the quality criteria for transplantation, after informed consent of the relatives, according to ethical approval granted by the local ethics committee [IRB of the Medical Faculty of the University of Wuerzburg; approval number 182/10 and 280/18sc]) were mixed with collagen extracted from rat tails. The mixture was transferred into the *trans* wells and compressed to a thickness of 500 µm. The models were initially cultivated in E1 medium (EpiLife with 1 µM calcium [Gibco], human keratinocyte growth supplements [HKGS; Gibco], and 1% penicillin and streptomycin) at 37°C with 5% CO_2_. On the following day, 5 × 10^5^ hTCEpi epithelial cells were seeded onto the stromal equivalent in E2 medium (E1 medium with 300 mM calcium chloride). After 24 h, the airlift was performed, raising the epithelium of the models above the surface of the medium. Over the next 12 days, the epithelium was grown into a multilayer in E3 medium (E2 medium with keratocyte growth factor [Gibco] and ascorbate-2-phosphate).

### Infection assays

Confluent epithelial monolayers were infected with conidia from FSSC keratitis isolates or *C. albicans* SC5314 yeast cells at a multiplicity of infection (MOI) of 1. The 3D hemi-cornea models, cultivated for two weeks, were infected with 1 × 10^5^ *Fusarium* spp. microconidia or *C. albicans* cells. Fungal cells were harvested as described above, and the concentration was adjusted according to the respective experiment. Infected monolayers or 3D models were incubated at 34°C and 5% CO_2_ for up to 48 h.

### Growth assay

Fungal conidia were harvested and quantified from Sabouraud agar plates as described before. A total of 1 × 10^5^ conidia in 10 µL sterile water were dropped on Sabouraud agar plates and incubated for up to 11 days at room temperature (approximately 22°C–23°C), 28°C, 34°C, or 37°C. Subsequently, the diameter and radius of radial fungal mycelium growth were measured.

### Adhesion assays

Confluent hTCEpi monolayers were infected as described before in 24-well plates with collagen I-coated coverslips. Cells were fixed with 4% paraformaldehyde after incubation for 20 min and 60 min at 34°C with 5% CO_2_. After five extensive washes with 1× PBS, the samples were stained with a 1:200 dilution of calcofluor white (Sigma Aldrich) and a 1:1000 dilution of phalloidin-rhodamine (Sigma Aldrich). After two washes with 1× PBS, coverslips were placed on microscopy slides with mounting medium containing Sytox DeepRed nuclear stain (Thermo Fisher). Microscopy was performed using a Nikon Ti2-E spinning-disc microscope (Nikon). An area of 2 mm^2^ was quantified per sample.

### Invasion assays

Confluent hTCEpi cells were infected with 1 × 10^5^ fungal conidia as described for the adhesion assays. After 6 h and 9 h of incubation at 34°C with 5% CO_2_, the cells were fixed with 4% paraformaldehyde. A 5% bovine serum albumin (BSA) blocking buffer was applied for 1 h at RT to prevent unspecific antibody binding. Cells were then incubated overnight at 4°C with a 1:100 dilution of primary mouse anti-*Fusarium* antibody (ISCA diagnostics) in 5% BSA blocking buffer. After two washes with 1× PBS, permeabilization was performed with 0.2% Triton X for 3 min. The cells were then incubated for 1 h at RT with a 1:500 dilution of secondary Alexa488-anti-mouse antibody (Thermo Fisher) in combination with a 1:1,000 dilution of Phalloidin-Rhodamine. Chitin was stained with a 1:200 dilution of calcofluor white (Sigma Aldrich) for 3 min and washed two times with 1× PBS afterward. Coverslips were embedded on microscopy slides with mounting medium containing Sytox DeepRed (Thermo Fisher). Microscopy was performed using a Nikon Ti2-E spinning-disc microscope (Nikon).

### Damage detection assay

Lactate dehydrogenase (LDH) release was measured using the Cytotoxicity Detection Kit (LDH) (Roche) according to the manufacturer’s manual. Briefly, 100 µL of cell supernatants were incubated with 100 µL of reaction mix for 30 min. The absorption was measured at a wavelength of 492 nm, with a background measurement at 640 nm, using a SpectraMax Id3 plate reader (Molecular Devices). Damage was calculated as relative values between the low control (uninfected hTCEpi cells or 3D models) and the high control (maximum damage in hTCEpi cells or 3D models treated with Triton X). Damage values were calculated in percentage of the damage detected for *C. albicans* which acted as a positive control in these assays.

### Cytokine assay

3D hemi-cornea models infected with FSSC isolates or *C. albicans* were incubated for up to 48 h. Cell media supernatants were collected 24 h and 48 h post-infection and stored at −80°C. The release of IL-6, IL-8, and TNF-α was measured using the ProcartaPlex Immunoassay (Thermo Fisher) according to the manufacturer’s .

### Histology

3D models or donor corneal tissue were fixed for 4 h with 4% paraformaldehyde and then embedded in paraffin after dehydration with xylol and isopropanol. Subsequently, the 3D models were deparaffinized. Sections 3 µm thick slices were cut using a microtome (Leica). The sections were placed on microscopy slides and deparaffinized for 1 h at 60°C using dry heat. Rehydration was carried out with xylol and ethanol, followed by 6 min of hematoxylin staining and 6 min of eosin staining. After the dehydration, the slides were mounted with Entellan (Sigma Aldrich) and dried overnight. Microscopy was performed using a Nikon Ni2 fluorescence microscope (Nikon). Stromal invasion was quantified in a rectangular area of approximately 0.1 mm^2^ using NIS Elements advanced research software (Nikon).

### Transmission electron microscopy (TEM)

Confluent hTCEpi cells were infected with 1 × 10^5^ conidia of FSSC keratitis isolates for 6 h. Cells were subsequently fixed with 2.5% glutaraldehyde and further processed as described previously (23). Histological cuts with about 80 nm thickness were analyzed with a JOEL JEM-1400 Flash Microscope (JOEL), operating at 120 kV with a Matataki Flash 2k × 2k camera system. The sections were cut horizontally through the FSSC-infected hTCEpi monolayer.

## RESULTS

To analyze the virulence attributes of FSSC species, we selected one clinical isolate each of *F. falciforme*, *F. keratoplasticum*, and *F. petroliphilum*, all of which were obtained from corneal swabs of keratitis patients (Table 1). Antifungal therapy with amphotericin B and voriconazole was performed for the two patients infected with either *F. keratoplasticum* or *F. petroliphilum*, while the antifungal treatment against the infection with *F. falciforme* was unknown (Table 1). Antifungal susceptibility was already previously tested, and the minimal inhibitory concentrations (MICs) were between 4 and 8 mg/L for azoles and 2 to 4 mg/L for amphotericin B and natamycin (2). Three further strains of *F. falciforme*, *F. petroliphilum*, and *F. keratoplasticum* from patients with keratitis, endophthalmitis, and systemic fusariosis were included (Table 1). The latter was not possible for *F. falciforme*, as there are no known cases of systemic infections in humans.

**TABLE 1:** Characteristics of FSSC keratitis isolate patients

FSSC isolate		Patient		Clinical data		
Species	NRZ no.	Age (yr)	Sex	Diagnosis	Origin of Isolation	Treatment
*F. falciforme*	2015-096	58	Female	Keratitis	Eye (not further specified)	Unknown
	2016-123	32	Female	Keratitis	Conjunctiva swab	Voriconazole
	2020-612	45	Female	Keratitis	Corneal swab	Voriconazole, amphotericin B
	2019-673	52	Female	Keratitis	Corneal swab	Unknown
*F. petroliphilum*	2014-079	20	Male	Keratitis	Corneal swab	Amphotericin B
	2014-013	56	Male	Keratitis	Isolate from eye chamber	Terbinafine, natamycin
	2017-410	38	Female	Endophthalmitis	Corneal swab	Amphotericin B
	2018-201	61	Unknown	Endocarditis	Isolate from aortic valve	Unknown
*F. keratoplasticum*	2014-069	60	Female	Keratitis	Corneal swab	Topical: amphotericin B, voriconazole Systemic: voriconazole
	2017-556	68	Female	Endophthalmitis	Unknown	Topical: natamycin Systemic: voriconazole
	2016-116	53	Female	Keratitis	Isolate from corneal disc	Topical and systemic: voriconazole
	2021-330	65	Unknown	Liver abscess	Liver puncture	Unknown
	2021-275	58	Unknown	Endocarditis	Isolate from blood culture	Unknown

### Growth kinetics of FSSC isolates at different temperatures

We compared the temperature-dependent growth dynamics of the three FSSC species and observed that at 34°C, the physiological temperature of the ocular surface, *F. petroliphilum* strains exhibited slower growth kinetics than the other species. The fastest growth for all species was observed at 28°C. None of the FSSC species grew at 37°C (Fig. S1).

### Clinical FSSC isolates show a low rate of adhesion to human corneal epithelial cells

**Fig 1:**
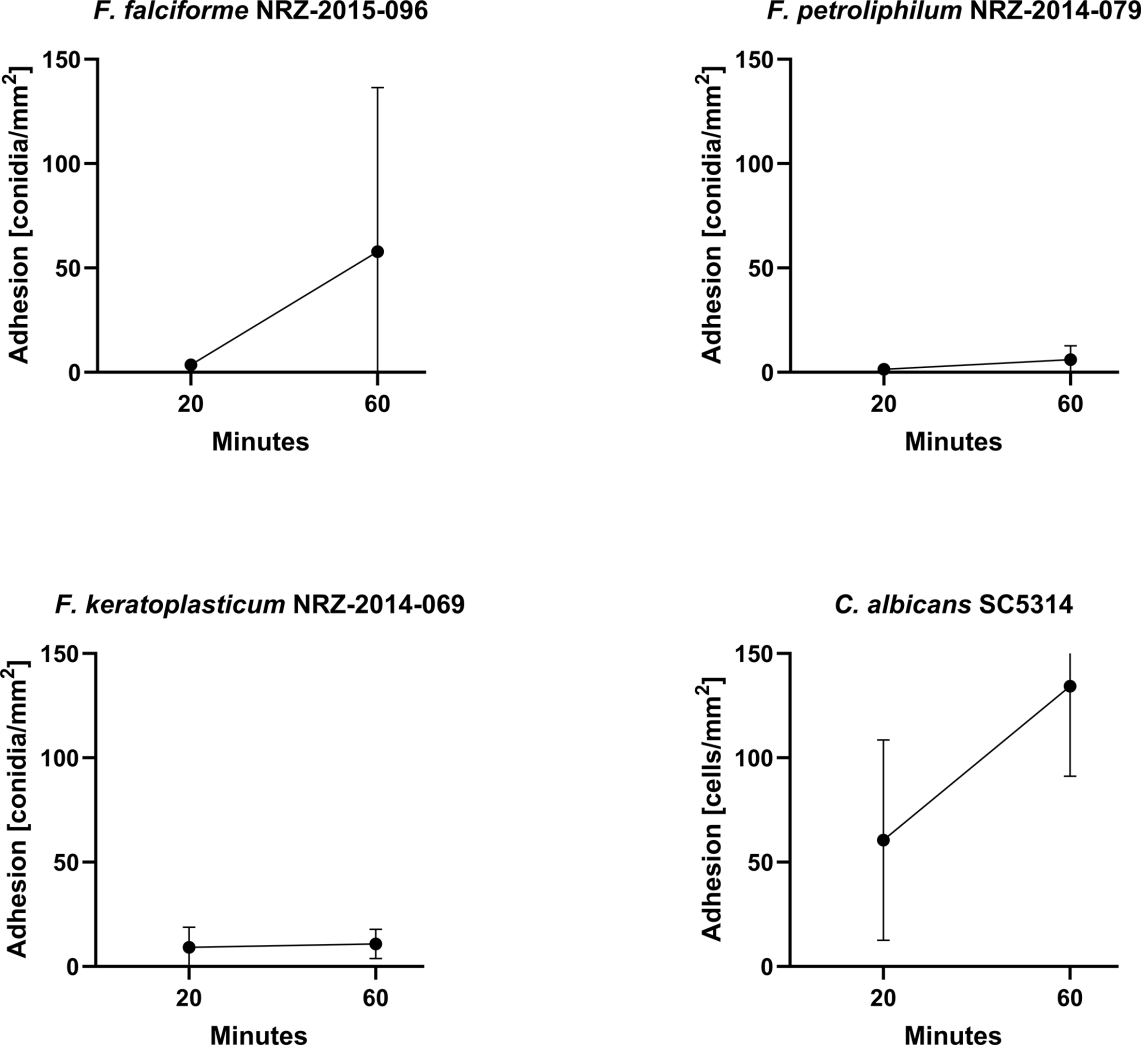
Adhesion of fungal cells to hTCEpi cells. The hTCEpi cells were incubated with fungal conidia for 60 min. All three tested FSSC isolates exhibited lower adherence compared to control strain *C. albicans* SC5314. Adherence was quantified based on the number of conidia adhering per square millimeter. The experiment was performed in triplicates.

The initial adhesion of *Fusarium* conidia to the human corneal epithelial hTCEpi cells was investigated in a 2D confluent monolayer infection model with a multiplicity of infection (MOI) of 1. *Candida albicans* SC5314 was used as a control. In accordance with previous work, host and fungal cells were co-incubated for up to 60 min, followed by five intensive washing steps to remove non-adhering conidia (24). As expected, *C. albicans* showed a high adhesion rate of 134 fungal cells/mm^2^. In contrast, *F. falciforme* had an adhesion rate of only 58 conidia/mm^2^ , and both *F. keratoplasticum* and *F. petroliphilum* conidia did not exceed 10 conidia/mm^2^ (Fig. 1).

### Hyphae of clinical FSSC isolates can invade human corneal epithelial cells

**Fig 2:**
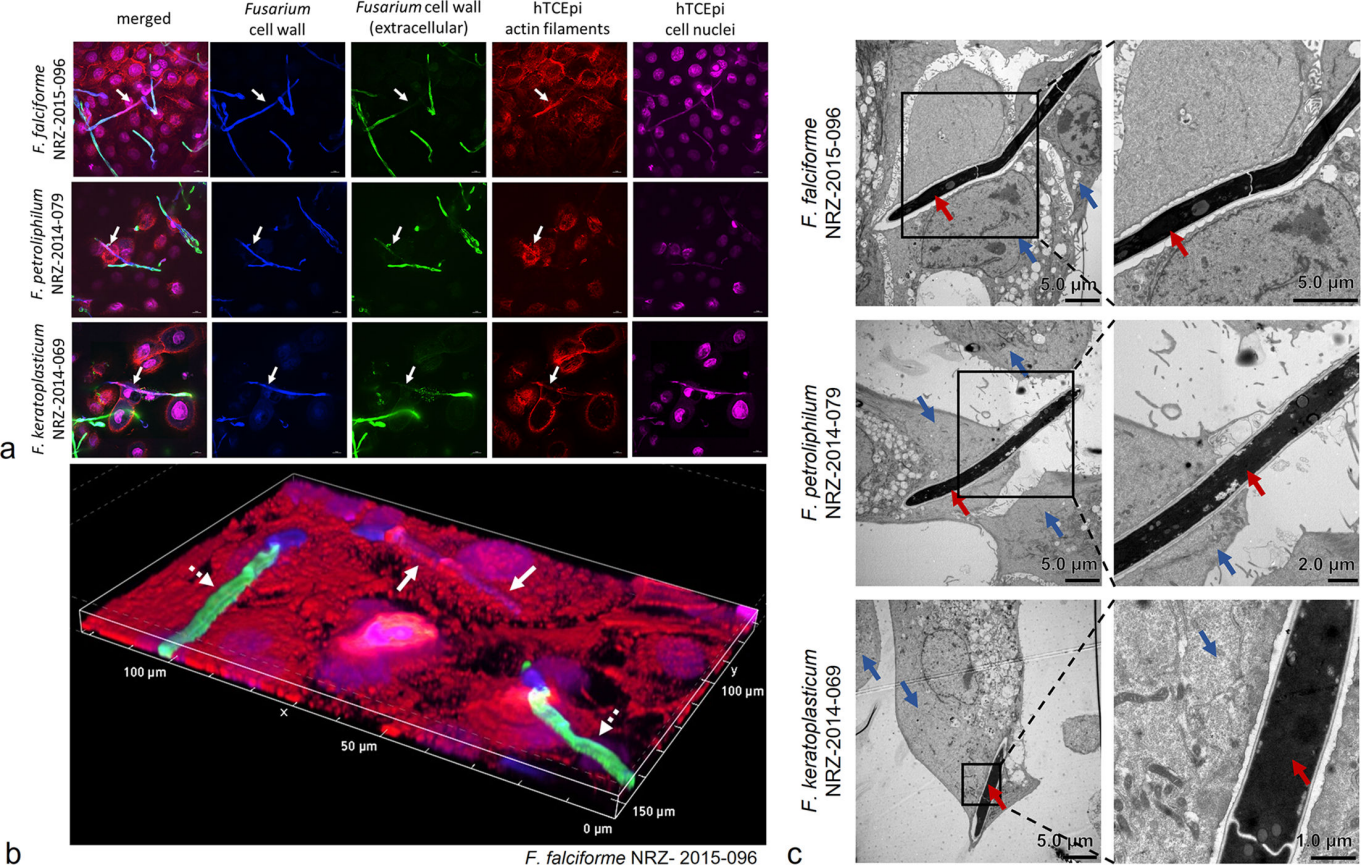
Invasion of FSSC keratitis isolates into monolayers of hTCEpi cells. (**a**) Differential staining of FSSC keratitis isolates on hTCEpi cells after 6 h of infection. While extracellular cell wall parts were double-positive for anti-*Fusarium* antibody (green fluorescence) and calcofluor white (blue fluorescence) staining, intracellular cell walls were only for positive calcofluor white. Host cell actin filaments were stained with phalloidin-rhodamine (red fluorescence) and cell nuclei with Sytox DeepRed (purple fluorescence). Intracellular hyphae with actin accumulation are indicated with white arrows. Magnification, 100×. (**b**) Z-stack imaging of *F. falciforme* infection on hTCEpi cells. Staining was performed as described in panel **a**. Dashed white arrows indicate extracellular hyphae with anti-*Fusarium* antibody and calcofluor white staining. Continuous white arrows point towards intracellular hyphae which are only calcofluor white-positive. Magnification, 100×. (**c**) Transmission electron microscopy (TEM) of FSSC isolates on hTCEpi cells. Red arrows indicate fungal hyphae, while blue arrows indicate hTCEpi cells, at 800× magnification. The right-hand panels display magnifications of the regions outlined in black in the corresponding left-hand panels.

Fungal adherence does not necessarily correlate with the potential to invade or damage host tissue (25). Thus, we further analyzed the invasion properties of the FSSC species. At 6 h post-infection, we examined hyphal internalization by corneal epithelial cells using differential staining of extra- and intracellular fungal parts, and invasion could be observed for all three FSSC species (Fig. 2a, white arrows). Occasionally, fungal hyphae were completely internalized (Fig. 2b, white arrows). Invading fungal hyphae were surrounded by host cell actin accumulation (Fig. 2a and b). Further analysis of fungal invasion into cornea epithelial cells by transmission electron microscopy revealed that *F. falciforme* and *F. keratoplasticum* hyphae partially spread across multiple cells (Fig. 2c, blue arrows). Quantification of a single experiment showed that the used *F. keratoplasticum* isolate was more capable to invade and displayed a much faster filamentation than examined isolates of *F. falciforme* and *F. petroliphilum* (Fig. S2).

### *Fusarium keratoplasticum* hyphae can induce the formation of transcellular tunnels

**Fig 3:**
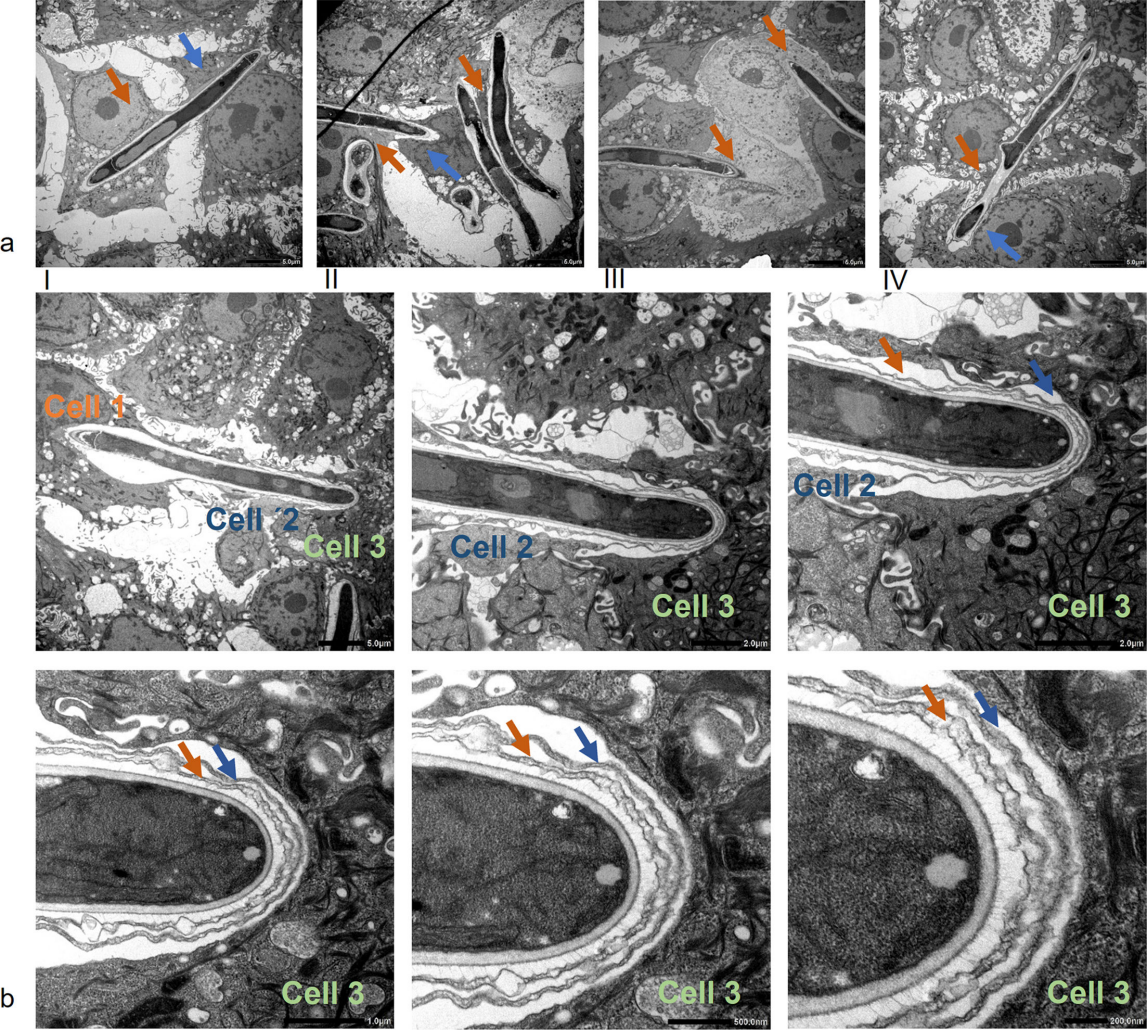
*F. keratoplasticum* hyphae can induce transcellular tunnel formation. Transmission electron microscopy (TEM) of *F. keratoplasticum* hyphae invasion routes into hTCEpi. (**a**) Different invasion routes observed at 800× magnification: invasion involving host membrane penetration (**I**), invasion by multiple hyphae per hTCEpi cell (**II**), invasion accompanied by breakdown of host cell (**III**), invasion without initial plasma membrane penetration, and characterized by formation of transcellular tunnels (**IV**). The first invaded host cell is marked with an orange arrow, while the second invaded host cell is marked with a blue arrow. (**b**) Transcellular tunnel formation during invasion in 800× up to 20,000× magnification. Invaded host cells are marked in orange, blue, and green. Arrows refer to the first (orange) and second (blue) layer of the surrounding cell membranes.

Dissemination of *F. keratoplasticum* hyphae within the infected monolayers was further analyzed by transmission electron microscopy (Fig. 3a). Sometimes, fungal hyphae were able to invade two neighboring cornea epithelial cells by penetrating their cell membranes during host cell entry (Fig. 3a I, blue arrow) and exit (Fig. 3a I, orange arrow). The host cells maintained their physiological structure during this mode of fungal invasion, even while being penetrated by multiple hyphae (Fig. 3a I to III). More interestingly, *F. keratoplasticum* hyphae could also disseminate throughout multiple host cells without penetrating host cell plasma membranes (Fig. 3a IV, orange, blue, and green arrow). In one instance, hyphae spread through three neighboring host cells without affecting the plasma cell membranes of the infected host cells (Fig. 3b). The cell membranes expanded significantly while still tightly engulfing the invading fungal hyphae, ultimately forming a structure that resembled transcellular tunnels (Fig. 3b). With each new expansion into an additional host cell, a new membrane layer was added (Fig. 3b, orange and blue arrow). Host cell nuclei appeared to be intact, indicating that infected cells were still alive (Fig. 3b). This behavior of *F. keratoplasticum* hyphae resembled the recently described phenomenon observed for *C. albicans* (26).

### Damage of *Fusarium* spp. on hTCEpi cells is species specific

**Fig 4:**
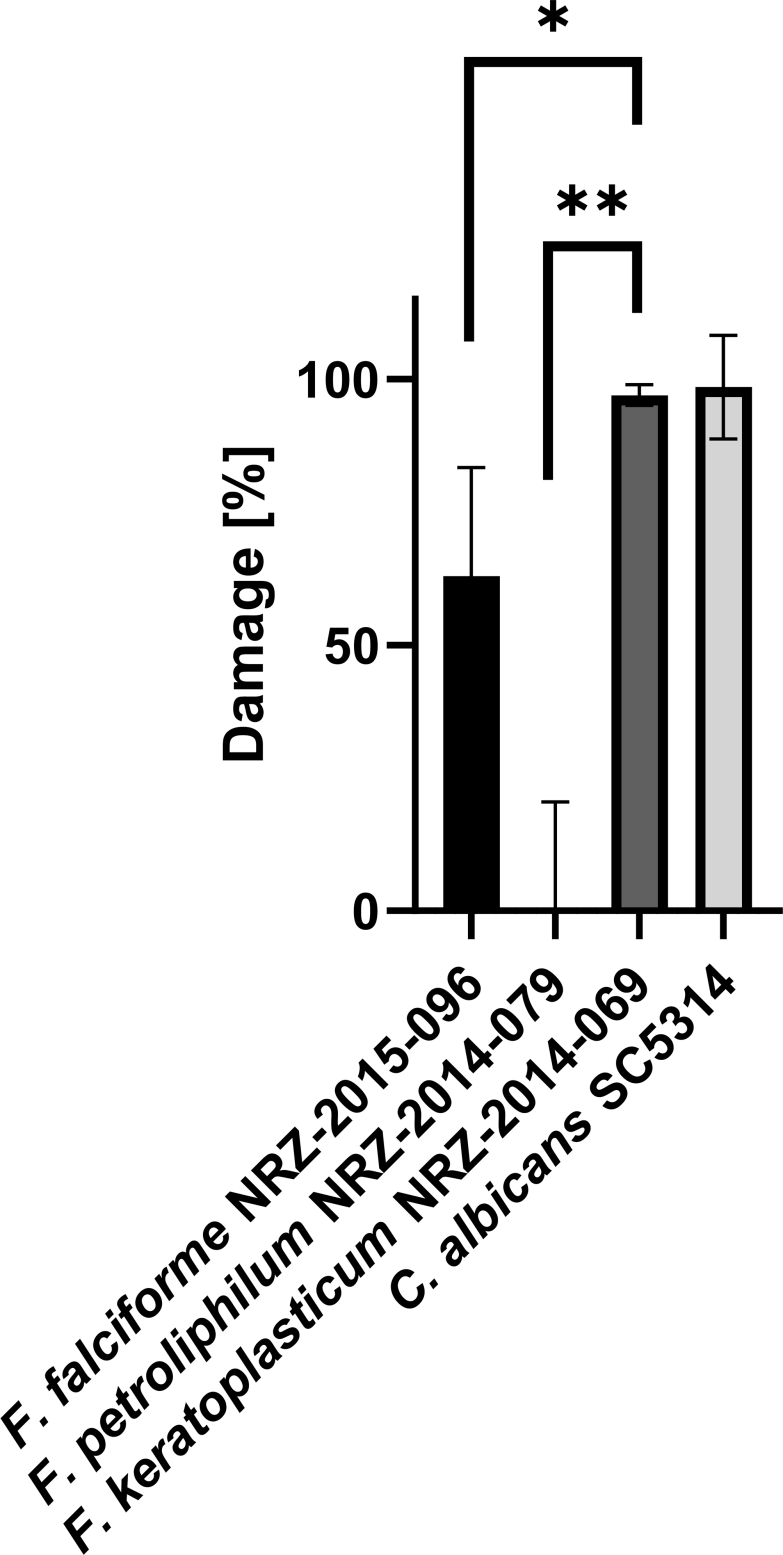
Damage of FSSC keratitis isolates on hTCEpi cells. Confluent hTCEpi cells were infected for 48 h with *Fusarium* keratitis strains and the control strain *C. albicans* SC5314 at a MOI of 1. LDH release was quantified from the supernatants. *F. keratoplasticum* caused significantly higher LDH release than the other two FSSC species. Calculations were performed with Graphpad Prism software and a parametric, two-tailed unpaired *t*-test with three biological replicates per condition (*P < .05 and **P < .01).

**Fig 5:**
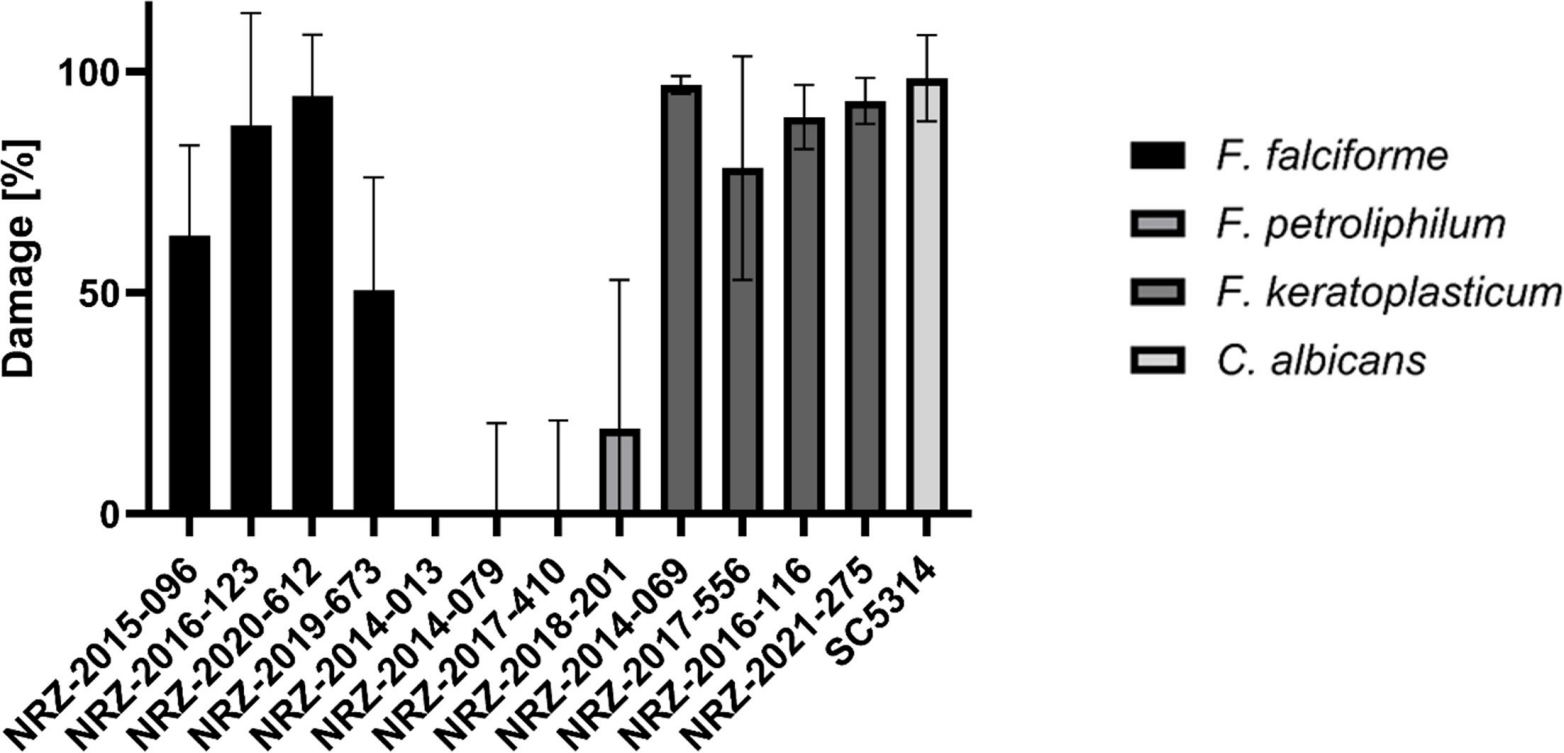
Damage of multiple FSSC keratitis and systemic infection isolates on hTCEpi cells. Confluent hTCEpi cells were infected for 48 h with *Fusarium* keratitis and systemic infection strains as well as the control strain *C. albicans* SC5314 at a MOI of 1. LDH release was quantified from the supernatants of infected cells (*n* = 3).

Finally, we investigated whether *F. falciforme*, *F. keratoplasticum*, and *F. petroliphilum* could cause host cell damage and used an established method to measure lactate dehydrogenase (LDH) release from host cells after 48 h of co-incubation with fungal cells. In accordance with its high invasion rate, *F. keratoplasticum* caused significantly higher host cell damage than *F. petroliphilum* and *F. falciforme* (Fig. 4). To exclude strain-specific effects, we extended the assay with three additional clinical isolates from each species. All *F. keratoplasticum* strains caused elevated cell damage, which was comparable to the control strain *C. albicans* SC5314 (Fig. 5). Interestingly, *F. falciforme* strains NRZ-2020-612 and NRZ-2016-123 were as damaging as *F. keratoplasticum* (Fig. 5). In accordance with the previous results, all *F. petroliphilum* isolates barely caused damage (Fig. 5).

### *Fusarium keratoplasticum* is the most invasive FSSC species in a 3D hemi-cornea model

**Fig 6:**
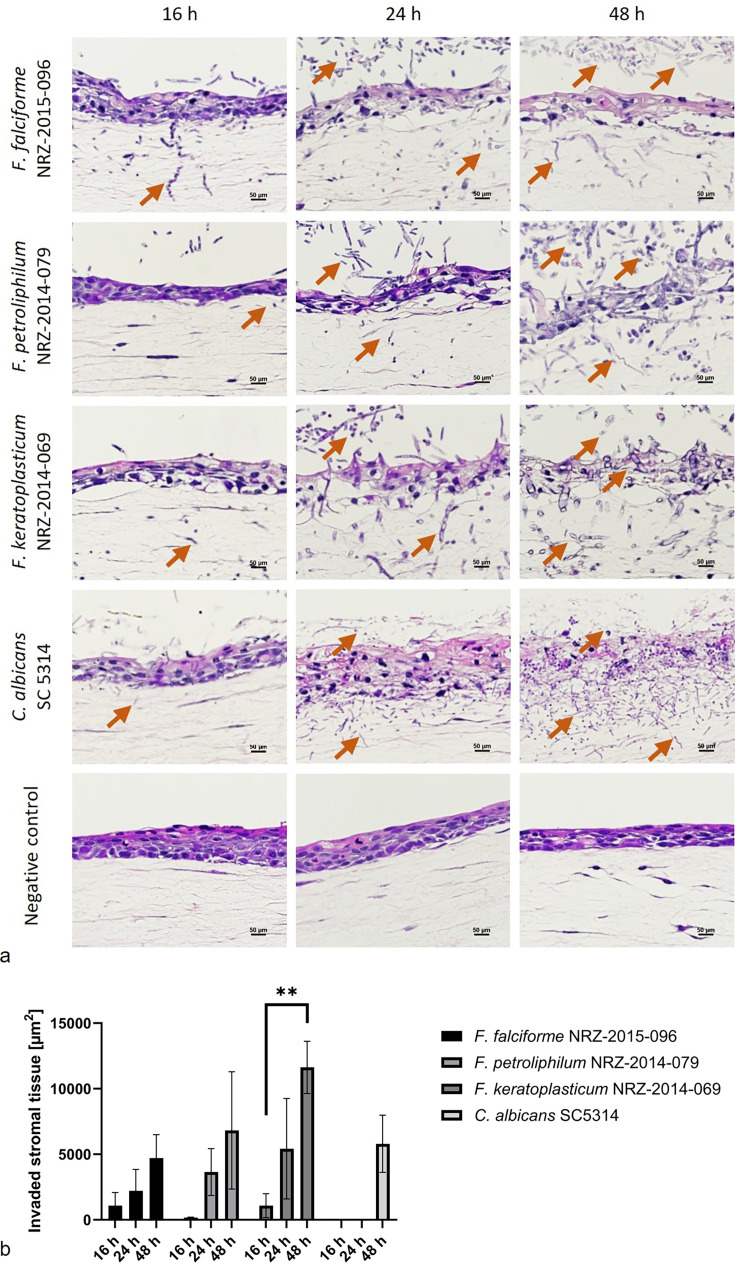
FSSC keratitis isolate invasion in a 3D hemi-cornea model. (**a**) Hematoxylin and eosin staining illustrates the time course of fungal invasion up to 48 h at 40× magnification. Orange arrows indicate fungal hyphae. (**b**) Quantification of fungal invasion into 3D model stroma cross sections. Invasive hyphae within a stromal area of approximately 100,000 µm^2^ (1,500 × 500 pixels) were evaluated at 16 h, 24 h, and 48 h post-infection as a percentage of the total stromal tissue (*n* = 3). Data were compared with a parametric, two-tailed, and unpaired *t* test (**P < .01).

Based on the observations for the 2D monolayer infection model, we proceeded to a more complex 3D hemi-cornea model which consists of a multilayer epithelium and a 500 µm thick stromal layer of collagen and fibroblasts, providing a much more accurate representation of the *in vivo* situation in keratitis compared to the 2D monolayer. The 3D models were infected with 1 × 10^5^ fungal conidia from the three FSSC species. Although fungal hyphae were detectable within the stroma 16 h after the infection, the epithelium was largely intact (Fig. 6a). The fungal burden increased to high density at 48 h post-infection, correlating with progressive degradation of the multilayer epithelium (Fig. 6a). At this time, fungal hyphae were also found within the stroma (Fig. 6a).

**Fig 7:**
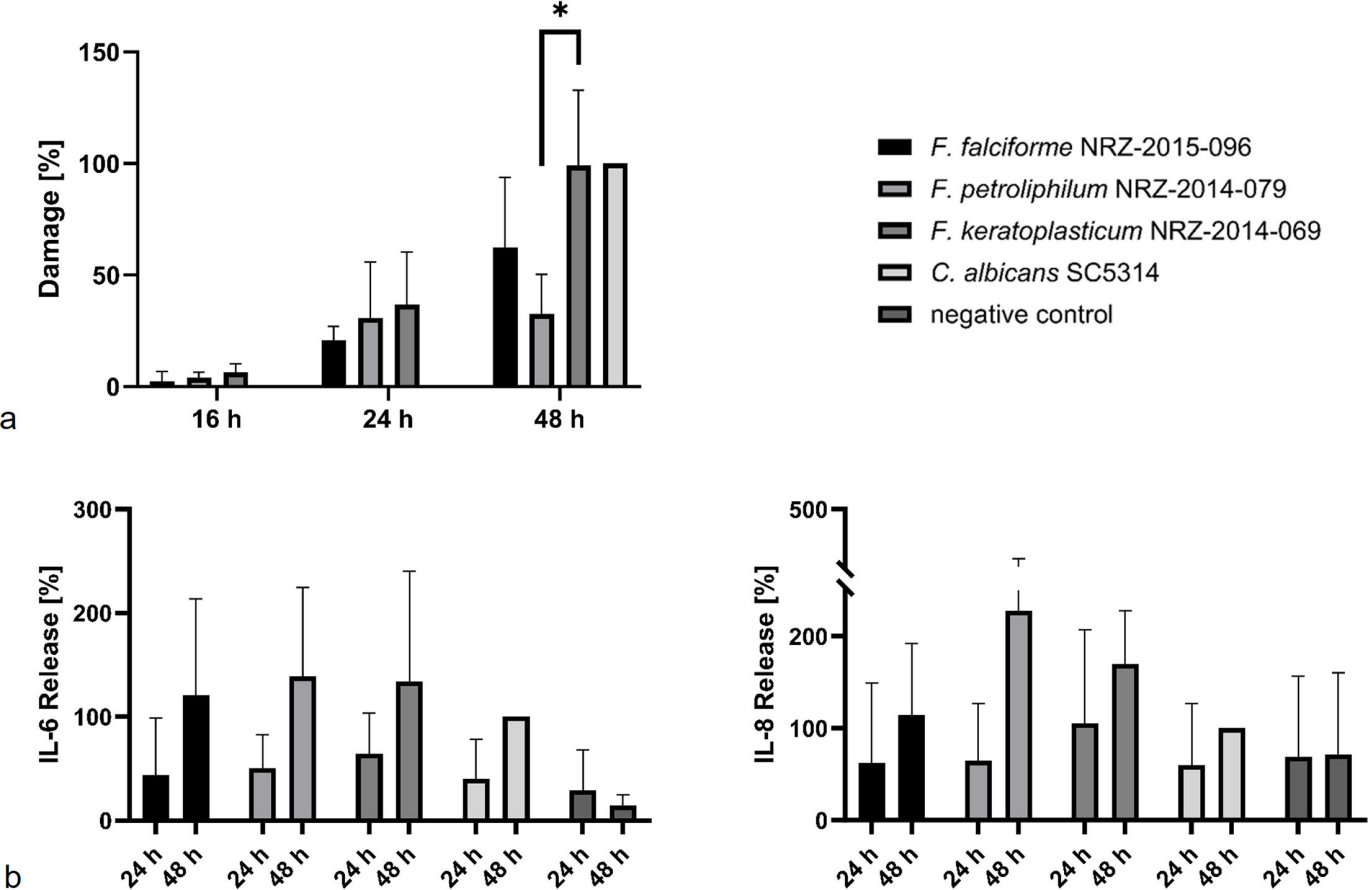
Damage and cytokine release in the *Fusarium*-infected 3D hemi-cornea model. Quantification of damage through LDH release (**a**) and cytokine release (**b**) was performed up to 48 h post-infection. Measurements were normalized relative to the positive control *C. albicans* SC5314 (*n* = 3). Asterisks indicate significant differences as calculated by using GraphPad Prism software and a two-tailed unpaired *t*-test (*P < .05).

To quantify fungal invasion, we determined the region of infected and non-infected stromal tissue within a total rectangle area of 100,000 µm^2^. *F. falciforme* and *F. keratoplasticum* were present in the stroma 16 h after infection of the 3D hemi-cornea models, and fungal invasion increased continuously over time (Fig. 6b). *F. falciforme* and *F. petroliphilum* invaded 4,701 µm^2^ and 6,817 µm^2^ of stromal tissue, respectively, at 48 h post-infection, while *F. keratoplasticum* reached an invasion area of 11,626 µm^2^ (Fig. 6b). It also caused the highest cell damage. While LDH release was very low 16 h post-infection, it increased over time and peaked at the 48 h (Fig. 7). Interestingly, cell damage caused by *F. keratoplasticum* was comparable to *C. albicans*, which was used for normalization, while *F. falciforme* and especially *F. petroliphilum* caused less damage (Fig. 7a). In addition, the release of the cytokines IL-6, IL-8, and TNF-α from host cells was measured. We did not observe a notable release of TNF-α, but all three FSSC species induced a higher release of IL-6 and IL-8 from infected hemi-cornea models than *C. albicans*, especially after 48 h of infection (Fig. 7b). However, it remained unclear whether these findings correlated with infection properties, as the lowly invasive *F. petroliphilum* triggered cytokine release levels to those of the highly invasive *F. keratoplasticum* (Fig. 7b).

## DISCUSSION

In this comparative pathogenicity study of *Fusarium solani* keratitis isolates, we identified specific infection properties, which were further highlighted by the advantages of our 3D infection model. Similar to *C. albicans*, FSSC infections of human corneal epithelial cells followed a pattern of adhesion, invasion, and destruction of the host cell. Although a comparison is difficult because the biology of yeast and resting conidia differs, we observed that FSSC conidia displayed a much slower adhesion rate than *C. albicans*. Given that eyelid blinking and the tear film contribute to a rapid mechanical elimination of non-adherent pathogens from the ocular surface (27), *Fusarium* cells must either adhere very quickly or rely on beneficial external conditions. The low adherence rate observed in our experiments rules out the first possibility and may explain why *Fusarium* keratitis is typically caused by external factors such as plant-derived injuries or the use of contaminated contact lenses. Once adhesion is successful, host cell invasion can occur. All three FSSC strains were found to be invasive, with their hyphae being intracellularly surrounded by host-derived actin accumulations, similar to the observations made for *C. albicans* hyphae (24, 25). In our settings, the used *F. keratoplasticum* strain was more invasive than the other FSSC isolates. In accordance with a previous report by Brown et al., we observed that invading FSSC hyphae were engulfed by host cell membranes (28). Surprisingly, in some cases, *F. keratoplasticum* hyphae induced the formation of transcellular tunnels through multiple host membrane layers, which had so far only been described for invading *C. albicans* hyphae on Caco-2 cells (24). It is important to note that these pathogen-induced tunnel formations do not cause cellular host damage, as the membrane layers remain intact. It might be beneficial during the progressing infection, as it could delay host defense responses and possibly facilitate the uptake of host cell metabolites (26). The transcellular tunnels induced by *F. keratoplasticum* also did not cause any breakdown of the membrane layers. Future experiments could determine whether this leads to a delayed host cell response to the invading fungal hyphae. The underlying cellular mechanisms that contribute to the high virulence potential of *F. keratoplasticum* remain unknown, but its adaptation to 34°C might play a crucial role. It has already been shown for the plant pathogen *F. graminearum* that resistance to heat stress strongly correlates with virulence (29). The specific pathogenicity of the FSSC keratitis isolates might also originate from differences in the ecological niche of the individual species. All three species were recorded from indoor environments, especially from sinks and drains. While *F. falciforme* has been frequently found in soil and a wide range of plants, *F. petroliphilum* was only isolated from cucurbits, and *F. keratoplasticum* has not been isolated from plants so far (5, 15, 16). Future experiments with more strains could help to evaluate species-specific infection routes and strategies.

Although the corneal epithelium is a crucial physical barrier against keratitis-causing pathogens, fungi commonly invade deeper tissue layers such as the stroma (9, 27). The cornea comprises five tissue layers with distinct functions. The stratified, non-keratinized epithelial layer serves as the main physical barrier against external factors, including microbes. It consists of five to six cellular layers with approximately 50 µm thickness. The Bowman’s layer separates the epithelium from the stroma, which makes up 90% of the total thickness (~500 µm) of the human cornea and mainly consists of collagen type-I and keratocytes. The Descemet’s membrane further separates the stroma from the monolayer endothelium (30). Given this complexity of the cornea, results from 2D monolayer infections offer limited insights into the progression of fungal keratitis. Therefore, more complex infection models for fungal keratitis often rely on animal infection models in mice, rabbits, and rats (21). Alternatively, recently developed 3D human cornea models allow the investigation of fungal infections under conditions that more closely resemble the physiology of the human cornea (31). To date, only Brown et al. have used a 3D stromal model for fungal keratitis infection, which consisted solely of the stromal layer (28). We used a 3D cornea infection model that combined the two main tissue layers—the multilayer epithelium and the stroma—thereby adding significant complexity to the infection model. Using this new model, we confirmed the findings from the 2D monolayer model that *F. keratoplasticum* was highly virulent, showing significant stromal invasion and causing a high host cell damage. The 48-hour time frame is comparable to that used in previous experiments using *ex vivo* human cornea models (32).

Based on our findings from two different infection models, we presume that *F. keratoplasticum* is well-adapted to infect human corneal epithelial cells, especially after the establishment of adhesion. Invasion may initially occur in a non-damaging manner, potentially by inducing transcellular tunnels, but ultimately lead to host cell destruction and dissemination into deeper parts of the cornea tissue. Interestingly, *C. albicans* exhibited a lower rate of stromal invasion after 48 h compared to *F. keratoplasticum*. In contrast to *Fusarium* filaments, *C. albicans* hyphae did not disseminate from the subepithelial layers into deeper parts of the model, resulting in different outcomes between the 3D and 2D models. In the latter, *C. albicans* appeared significantly more virulent. This highlights how the increased complexity of the 3D model provides a more comprehensive representation of the physiological infection process. Differences in the depth of stromal tissue penetration between *Fusarium* and *Candida* may reflect varying levels of accessibility to antimycotic treatments. Limited bioavailability and deep tissue penetration remain persistent obstacles in the management of fungal keratitis (33). Our model can be applied to more complex experiments, e.g., to identify the mechanisms by which FSSC species induce IL-8 secretion and consequently promote neutrophil migration into the infected tissue. Neutrophils are required for fungal clearance, but they also contribute to tissue damage by secreting metallopeptidases. A combined approach of the 3D model with neutrophils and available patient data might be helpful to elucidate if *F. keratoplasticum* virulence in the model correlates with poor prognosis for the patient.

Our study initiates an important step toward the elucidation of the complex infection process of *Fusarium* keratitis, ranging from initial epithelial adhesion to terminal stromal damage. We identified strain-specific invasion routes and demonstrated that *F. keratoplasticum* exhibits greater virulence compared to *F. falciforme* and *F. petroliphilum*. The newly established 3D infection model enabled us to study *Fusarium* keratitis in a more physiologically relevant context, which is crucial to translate research findings into concrete future benefits for patients.

## SUPPLEMENTAL MATERIAL

Figure S1 msphere.00328-25-s0001.tif Growth of FSSC keratitis isolates at different temperatures.

Figure S2 msphere.00328-25-s0002.tif FSSC hyphal characteristics in one invasion experiment with hTCEpi cells.

Supplemental Figure Legends msphere.00328-25-s0003.docx Legends to Figures S1 and S2.

ASM does not own the copyrights to Supplemental Material that may be linked to, or accessed through, an article. The authors have granted ASM a non-exclusive, world-wide license to publish the Supplemental Material files. Please contact the corresponding author directly for reuse.
