# Supplementary figures and images for: A 3D Fusarium keratitis model reveals isolate-specific adhesion and invasion properties in the Fusarium solani species complex
Source: mSphere. 2025 Nov 4;10(11):e00328-25. doi: 10.1128/msphere.00328-25 (PMC12646002; doi:10.1128/msphere.00328-25)

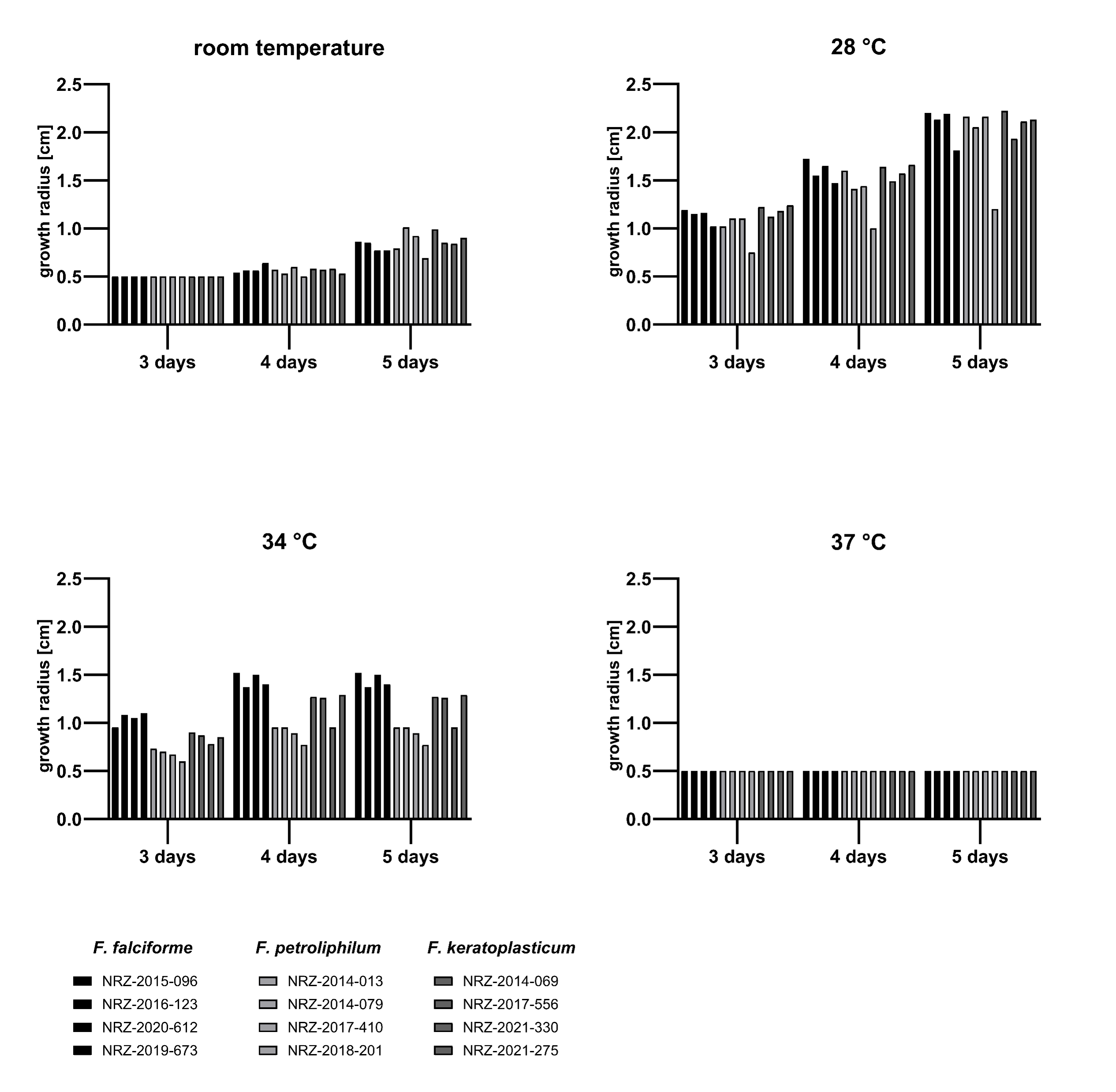

Supplement: Figure S1 — Growth of FSSC keratitis isolates at different temperatures. [file msphere.00328-25-s0001.tif]

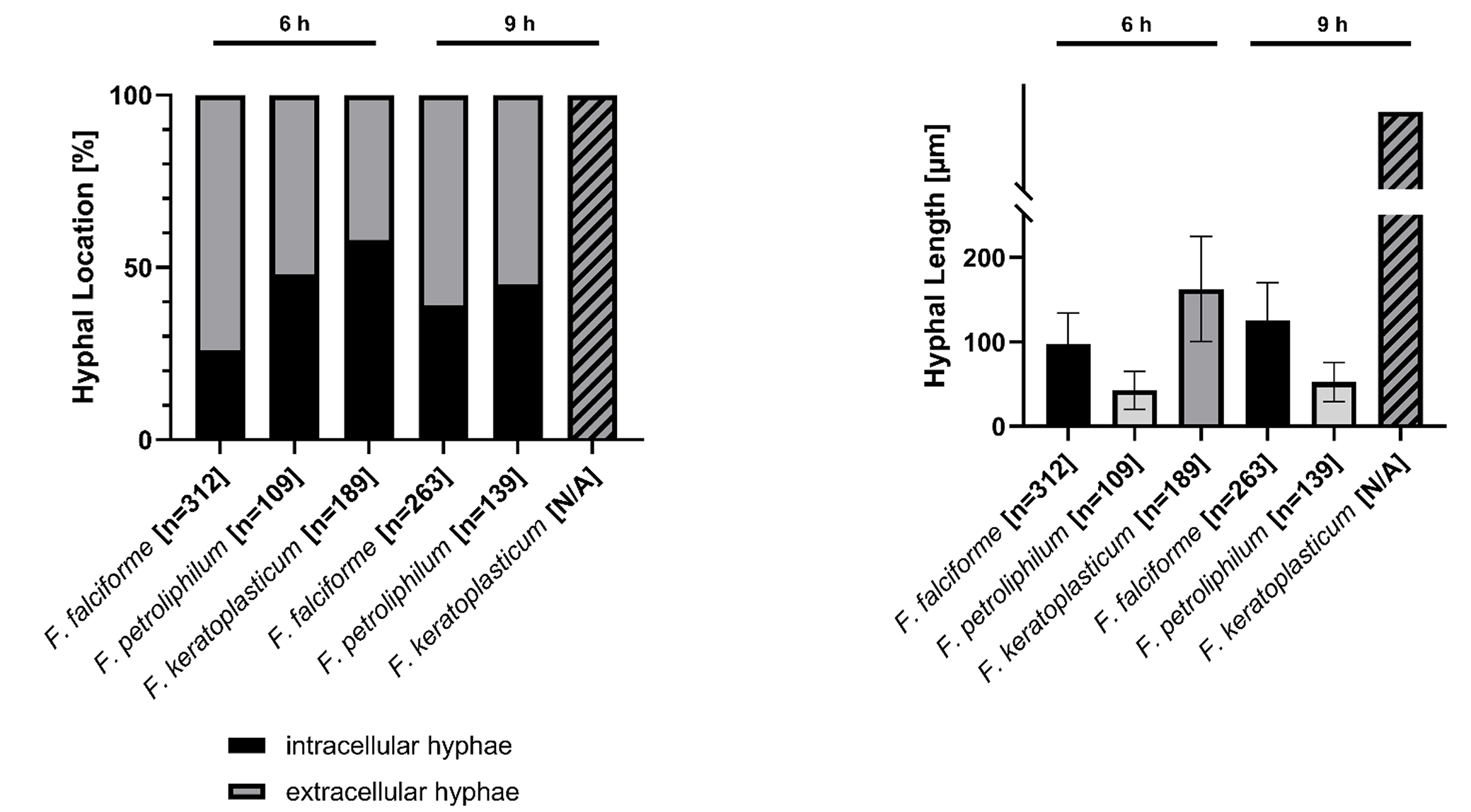

Supplement: Figure S2 — FSSC hyphal characteristics in one invasion experiment with hTCEpi cells. [file msphere.00328-25-s0002.tif]
