# Supplementary material for: A 3D Fusarium keratitis model reveals isolate-specific adhesion and invasion properties in the Fusarium solani species complex
Source: mSphere. 2025 Nov 4;10(11):e00328-25. doi: 10.1128/msphere.00328-25 (PMC12646002; doi:10.1128/msphere.00328-25)
Supplement: Supplemental Figure Legends — Legends to Figures S1 and S2. [file msphere.00328-25-s0003.docx]

**Supplementary Figure S1. Growth of FSSC keratitis isolates at different temperatures.**

1x10^5^ fungal conidia were plated as centrical drop on sabouraud agar plates and diameter of growth was measured at different times. Four clinical strains per species were used, and growth was observed over a period of 5 days. The highest growth was observed at 28 °C. *F. petroliphilum* strains exhibited reduced growth at 34 °C compared to the other species.

**Supplementary Figure S2. FSSC hyphal characteristics in one invasion experiment with hTCEpi cells.**

Based on pictures as shown in Figure 2A, hyphal invasion events and hyphal lengths were quantified after 6 h and 9 h of infection. Hyphal invasion was assumed when actin accumulation was observed around hyphae in the absence of antibody staining in differential fluorescence stainings. Invasion rate and hyphal length were calculated within a rectangular area of 1 mm^2^ using NIS Elements Advanced research software (Nikon). Between 109 to 312 hyphae were counted per isolate within one biological experiment.
